# Influence of the Maxillary Sinus on the Accuracy of the Root ZX Apex Locator: An Ex Vivo Study

**DOI:** 10.3390/dj7010003

**Published:** 2019-01-02

**Authors:** Roula El Hachem, Elie Wassef, Nadim Mokbel, Richard Abboud, Carla Zogheib, Nada El Osta, Alfred Naaman

**Affiliations:** 1 Department of Endodontics, Faculty of Dentistry, Saint Joseph University, P.O. Box 11-5076 Riad el-Solh, Beirut 1107 2180, Lebanon; carla.zogheibmoubarak@usj.edu.lb (C.Z.); alfred.naaman@usj.edu.lb (A.N.); 2 Department of Periodontics, Faculty of Dentistry, Saint Joseph University, P.O. Box 11-5076 Riad el-Solh, Beirut 1107 2180, Lebanon; elie.wassef@usj.edu.lb (E.W.); nadim.mokbel@usj.edu.lb (N.M.); 3 Department of Maxillo-Facial Radiology, Saint Joseph University, B.P. 11-514 Riad el-Solh, Beirut 1107 2050, Lebanon; Richard.abboud@usj.edu.lb; 4 Department of Prosthodontics, Saint Joseph University, B.P. 11-514 Riad el-Solh, Beirut 1107 2050, Lebanon; nada.osta@usj.edu.lb

**Keywords:** maxillary molar, maxillary sinus, working length, Root ZX, apex locator

## Abstract

This study evaluated the accuracy of the Root ZX (J. Morita, Tokyo, Japan) electronic apex locator in determining the working length when palatal maxillary molar roots are in a relationship with the sinus. Seventeen human maxillary molars with vital pulp were scheduled for an extraction and implant placement as part of a periodontal treatment plan. The access cavity was prepared, and a #10 K file (Dentsply Maillefer, Ballaigues, Switzerland) was inserted into the palatal root using the Root ZX apex locator in order to determine the electronic working length (EWL); then, the teeth were extracted. To determine the real working length (RWL), a #10 K file was introduced into the root canal until its tip touched a glass plate. EWL and RWL were compared. Images reconstructed with CBCT (cone beam computerized tomography) revealed that eight palatal roots were related to the maxillary sinus, whereas nine were not. The results showed a significant difference between the EWL and the RWL of the palatal roots related to the sinus (*p* < 0.001). No significant difference was observed in measurements of roots not in contact with the sinus (*p* > 0.05). Within the study limitations, the reliability of Root ZX was influenced by the relationship of the roots with the maxillary sinus.

## 1. Introduction

The elimination of microorganisms and pulp tissue from the root canal system is a crucial step for endodontic success [1,2], and such treatment should be confined within the roots [3,4]. This can only be achieved when the root canal length is determined with accuracy. Any endodontic act beyond or below this limit may increase the risk of failure due to overfilling or underfilling [5]. It is sometimes tricky to precisely determine the root canal preparation length using periapical radiographs, since they are subject to distortion, are sensitive in interpretation, and they only give a two-dimensional image of the three-dimensional roots [6].

The introduction of electronic apex locators (EAL) has allowed better reliability in working length (WL) determination [7,8] while minimizing the exposure of patients to radiation [9]. The latest generation of these devices determines the WL by measuring the variation of impedance by using different frequencies in the instrument tip within the canal [10]. Root ZX (J. Morita, Tokyo, Japan) is an accurate, third-generation EAL that expresses the quotient between the impedances of two frequencies (0.4 and 8 kHz) and indicates the position of the instrument inside the root canal. It ensures high levels of efficiency and accuracy, making it the gold standard EAL [11,12,13]. However, some conditions might affect its performance. The influence of various factors on the accuracy of Root ZX has been studied, such as the degree of root canal curvature [14], tooth group [15], apical foramen diameter and file size [16], pulp vitality [17], presence of irrigants [18], and endodontic perforation [19]. Piasecki et al. [20] found that EAL performance may be affected by apical anatomic complexities. However, the effect of root apices being in contact with the maxillary sinus on the reliability of EAL has not been studied.

The maxillary sinus is an anatomic organ that is localized near the nasal cavity in close proximity to the upper molars [21]. Crucial care should be taken during surgeries [22,23] and endodontic therapy on molars with roots in close proximity to the maxillary sinus [24]. Related to this, Matsumoto et al. [25] showed that odontogenic infection was the cause of 70% of 190 studied unilateral sinusitis cases. The occurrence of iatrogenic errors may induce sinusitis and infections resulting from thickening of the maxillary sinus mucosa [26] or the extrusion of endodontic files, irrigation solutions, or sealers into the sinus cavity while performing cleaning and obturation of the root canal system [27,28].

Cone beam computerized tomography (CBCT) imaging is an advanced, indicative imaging technique that has been adopted in root canal treatment for the three-dimension study of the root canal anatomy [29,30,31,32], as well as the evaluation of root resorption, the diagnosis of root fracture [33], and the determination of presurgical strategies [34,35]. It enables the topographic relationship between the sinus floor (MSF) and the maxillary molar roots to be established [36,37,38]. According to Aksoy and Orhan [39], in 84% of the second molars and in 77% of the first molars, at least one of the roots protrudes into the sinus or is in contact with it. Gu et al. [21] reported that maxillary molars are closer to the MSF than premolars and that age has a significant influence on the relationship between upper molars and the MSF. CBCT represents an important modality with great potential for clinical practice and precision compared to conventional radiography [40,41]. CBCT imaging offers several advantages over medical computed tomographic imaging, including its ease of use for dental applications, reduction of radiation doses, and lower cost [42,43,44,45].

To date, no studies have investigated the effect of apical root canal sinus projection on EAL accuracy. Considering the importance of the correct determination of WL when treating the upper molars, the relationship between the maxillary sinus and the roots of the maxillary posterior teeth, and to avoid endodontic complications of the sinus tissues, the aim of this ex vivo study was to evaluate the reliability of Root ZX when root canals are in contact with the maxillary sinus. The null hypothesis tested was that there are no significant differences in the reliability of the Root ZX between the roots whether they are in contact or not in contact with the maxillary sinus.

## 2. Materials and Methods 

This study was approved by the Ethics Committee of Saint Joseph University Beirut (USJ-2018-10) on 20 February 2018.

### 2.1. Selection of Teeth

Patients in good general health aged 50 to 60 years old were recruited from the Dental Health Care Center of Saint Joseph University of Beirut. They were scheduled for the extraction of an upper molar and placement of an implant as part of their periodontal treatment plan. After clinical and radiographic examinations, only maxillary molars with vital pulp and no symptoms of pulpitis were included in the study. Molars with open apices, fractures, root resorptions, calcifications, or previous endodontic treatments were excluded. Seventeen palatal roots were finally included in this study and written informed consent was obtained from the participants. 

### 2.2. Imaging Procedures and Evaluation of the CBCT Images

**Figure 1:**
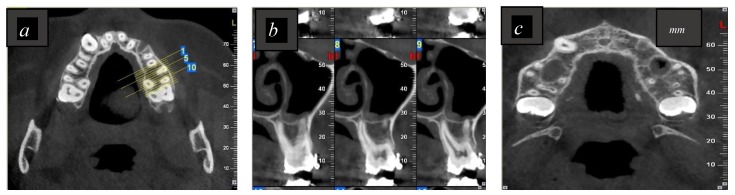
Representative cone beam computed tomography (CBCT) images showing the palatal root apex not in contact with the sinus floor (MSF). (**a**,**c**) A maxillary axial CBCT view; (**b**) a maxillary sagittal CBCT view.1; 5 and 10 are the slices numbers.

**Figure 2:**
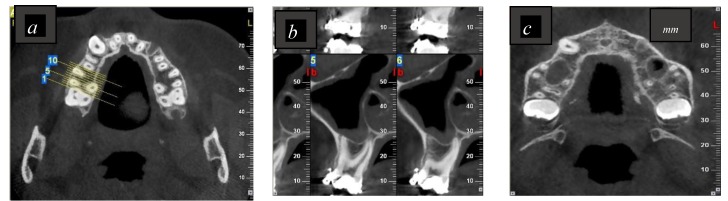
Representative CBCT images showing the palatal root apex in contact with the MSF. (**a**,**c**) A maxillary axial CBCT view; (**b**) a maxillary sagittal CBCT view. 1; 5 and 10 are the slices numbers.

Preoperatively, all patients received limited CBCT imaging as part of preimplant planning. The imaging system used was the NewTom VGI (QR, Verona, Italy) cone beam computed tomography (CBCT) with the following parameters: 3.3 mA, 110 kVp, 7.5 × 12 cm scan field of view, and 0.150 mm voxel size. The CBCT images were established by employing OnDemand3D software (Cybermed, Seoul, Korea) concordant to the operating parameters. Images were examined by an experienced oral radiologist in order to confirm whether or not the palatal root was in contact with the sinus, as shown in Figure 1 and Figure 2.

### 2.3. Determination of the Electronic Working Length Using Root ZX

The determination of the working length of the palatal root was completed by one endodontist who was blinded to the results of the CBCT. After applying local anesthesia and placing the rubber dam, an access cavity was done on each tooth using a handpiece at high speed. The reference point was standardized by flattening the palatal cusp tip. The Root ZX apex locator then determined the EWL in accordance with the manufacturer’s instructions. A #10 K file (Dentsply Maillefer, Ballaigues, Switzerland) was connected to the file clip of the EAL and inserted apically into the canal until the “Apex mark” appeared. The silicone stop was then cemented to the file with light-cured glass ionomer cement (GC Fuji^®^ Automix LC, GC Corp., Tokyo, Japan), and the distance between the stop and the file tip was measured with a digital caliper at 5× magnification (Carl Zeiss GmbH, Oberkochen, Germany). The working length obtained for each canal was then recorded and noted as the EWL. The canal was irrigated with 2.5% sodium hypochlorite (NaOCl), the rubber dam was removed, and an oral surgeon extracted the tooth and checked it to ensure that the root tips were not broken. A solution of 2.5% NaOCl was used to disinfect the root surface, and then the teeth were saved for 20 minutes in a saline solution in order to measure their real working lengths. 

### 2.4. Determination of the Real Working Length Using a Glass Plate

The real working length (RWL) of the palatal root was measured using a glass plate tangential to the plane of the anatomic foramen, and a #10 K-file (Dentsply Maillefer, Ballaigues, Switzerland) was introduced into the root canal until its tip touched the glass plate. The file was then cemented to the silicone stop using light-cured glass ionomer cement (GC Fuji^®^ Automix LC, GC Corp., Tokyo, Japan) in order to prevent movement. The file was then extracted, and the measurement was taken between the stop and the file tip. The endodontist applied the same technique as in EWL in order to measure distances. The WL obtained for each canal was then recorded and noted as the RWL. 

### 2.5. Statistical Analyses 

Statistical analyses were performed using the Statistical Package Software for Social Science (SPSS for Windows, Version 17.0, Chicago, IL, USA). The level of significance was set at *p* < 0.05. For each tooth, EWL and RWL were recorded three times, and the average measurement was calculated and used for the statistical analysis. The WL error was calculated by subtracting the EWL from the RWL (Error = EWL − RWL). Negative and positive values indicated measurements that were short and long of the real length, respectively, whereas 0.0 indicated coinciding measurements. The accuracy of the WL measurements was assessed using Student *t*-tests to compare the mean error between roots in contact and roots with no contact with the sinus. Paired Student *t*-tests were also executed to compare the mean EWL and RWL values for palatal roots in contact or not in contact with the sinus. One-sample *t*-tests were used to compare the mean error value with the theoretical value, 0, which assumes the absence of error.

## 3. Results

**Figure 3:**
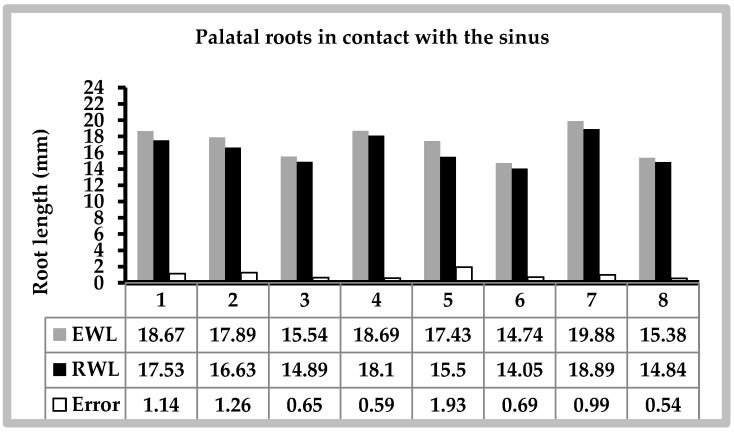
Electronic working length (EWL) versus real working length (RWL) of palatal roots in contact with the sinus.

**Figure 4:**
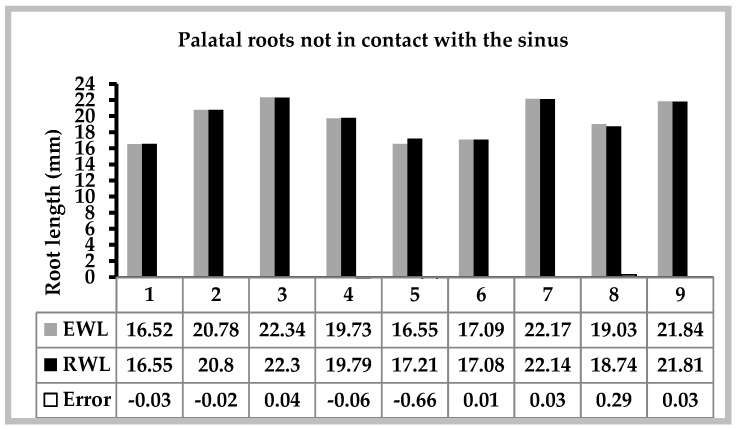
EWL versus RWL of palatal roots not in contact with the sinus.

Seventeen palatal roots were finally included in this study. The CBCT images showed that eight were in contact with the sinus and nine were not. A comparison of the EWL versus the RWL of palatal roots related to or not related to the sinus are detailed in Figure 3 and Figure 4. The mean measurements and standard deviations obtained in groups are shown in Table 1. There were no significant differences in the EWL and RWL when the roots were not in a relationship with the sinus (*p* = 0.744). However, the mean EWL was significantly higher than RWL for roots related to the sinus (*p* = 0.001). Moreover, palatal roots in relation with the maxillary sinus had a mean error that was significantly different from 0 (+0.974, *p* = 0.001). However, the mean error was not statistically significant for palatal roots with no sinus contact (−0.041, *p* = 0.639).

## 4. Discussion

The aim of this study was to evaluate the reliability of the Root ZX locator when root canals are in contact with the maxillary sinus. To our knowledge, the effect of the relationship between the root apex of upper molars and the maxillary sinus on EAL accuracy has not been studied previously.

Recently, different studies have reported the exactitude of several EALs [14,46,47,48]. In this current ex vivo study, one of the critical steps in this process was the standardization of samples. We used the palatal roots of the first and second maxillary molars, and all teeth were flattened to allow standardization and the creation of a flat reference point for accurate measurements [49]. Each tooth was isolated with a rubber dam to obtain a stable electronic reading [50]. All specimens were measured by the same Root ZX locator, as well as by the glass plate method. In addition, patients were of similar age, which provided comparable root growth and prevented morphological divergences.

Although Tsesis et al. [51] showed that EALs are not influenced by the pulp status, this study only consisted of teeth with vital pulp tissue in order to remove any potential bias. The Root ZX locator was used in accordance with the manufacturer’s instructions with a size #10 K file [52]. Although Nguyen et al. [53] claimed that the accuracy of EALs is not affected by the file size, the same file size was used in all canals to provide similar conditions for EWL and RWL. In this report, in contrast to the study conducted by Azabal et al. [54], the teeth were not decoronated; this kept the investigation variables nearest to an in vivo condition. Furthermore, the determination of the RWL was made when the #10 K file reached firm contact with the glass plate, instead of only using visual magnification; this gave a reduced margin of error. 

In the present study, all CBCT images were taken before the study, and no additional X-rays were done to reduce radiation. According to the classification of vertical relationships between the root apex of the second molars and the MSF, the frequency of a root projection in the sinus varies between 14% and 23% [51,55]. Wallace [56] reported that in 40% of cases, the first and the second maxillary molars communicate with the sinus. In this study, the CBCT taken as preimplant treatment planning was also used to assess whether the palatal maxillary molar roots were in contact with the sinus. The CBCT axial and sagittal images clearly showed that eight roots were associated with the sinus and nine were not.

This research showed a significant difference between the EWL and the RWL of the palatal roots in relation with the sinus, but did not demonstrate a significant difference in measurements with roots that were not in contact with the sinus. The EWL was revealed to be significantly longer than the RWL when the palatal roots were in contact with the sinus. Previous investigations showed overestimated measurements in 2.56% [12], 7.9% [57], and 3.1% [58] of cases, respectively, when using the Root ZX. However, in one study that analyzed WL determination, the use of Root ZX was found to significantly reduce the incidence of working length overestimation compared to periapical radiographs [59]. One explanation for the results of the current study may be that roots in contact with the maxillary sinus have different levels of impedance than roots not in contact with the sinus. This finding is crucial, since it demonstrates the risk of over instrumentation, especially for those endodontists who only use the EAL to accomplish their root canal treatment. This is clinically important as inaccurate determination of the WL when treating teeth with close proximity to the maxillary sinus may induce the extension of bacteria, endodontic tools, intracanal solutions, and root canal fillings into the sinus [36]. The introduction of necrotic debris and microorganisms into the sinus could cause chronic sinusitis [60]. Kang et al. [37], who found a close distance between the root apexes of upper molars and the MSF and procured approximate measurements from the root apexes to the buccal cortical plate, stressed the importance of giving appropriate attention when treating upper molars. In addition, overestimated WL can be accompanied by microbial contamination, injury to the periapical tissues, and pain, reducing the success rate of root canal therapy [3,61]. Correct bacterial disinfection, pertinent shaping, and three-dimensional obturation of the root canal system rely on the precise determination of the root length [62,63].

The Root ZX locator is considered a reference to which other EALs are compared [64], and several investigations have evaluated its reliability [65,66]. In the current study, for teeth not in contact with the sinus, there was no significant difference between the EWL and RWL. This is in accordance with results from other authors, who measured an accuracy level of Root ZX from 75% to 97.37% [67,68,69]. This device provides the WL by measuring the impedance with two frequencies between the file and the canal fluid without adjustment or calibration. This is the reason that Root ZX is considered to be highly precise in locating the apical foramen [58,70]. However, some previously conducted studies showed that there are several factors that can adversely influence the results [71,72] or even prohibit accurate WL determination, such as blood [73], the protocol adopted [10], the preoperative pulp status [74], the irrigant used [75], or the presence of open apices [62]. This study showed that the reliability of Root ZX is also influenced by the relationship of the root with the maxillary sinus.

There may be some limitations in this investigation. Primarily, the number of specimens was rather low, since strict inclusion criteria were applied. Even though the result of the power test was high, clinical studies over a bigger number of roots with a different degree of clinical situations are necessary to further confirm these findings.

Thus, the present study data rejected the null hypothesis that there would be no difference between the roots in contact with the sinus or below it in terms of the reliability of the Root ZX apex locator.

## 5. Conclusions

Despite the limitations of this study, a significant difference in the accuracy of the Root ZX apex locator between roots in contact or not in contact with the maxillary sinus was found. Realizing the anatomical relationship between the maxillary posterior teeth and the sinus, the clinician must be particularly cautious to prevent possible complications from root canal treatment involving maxillary posterior teeth. Therefore, it is recommended that periapical radiographs are combined with apex locators to determine an acute and reliable working length to allow the long-term success of the root canal treatment.

## Figures and Tables

**Table 1:** Mean and standard deviation measurements (in mm) of differences between the EWL and the RWL.

Groups	Mean RWL (mm)	Mean EWL (mm)	Mean Error
Roots in contact with the sinus (*n* = 8)	16.304 ± 1.748	17.278 ± 1.858	+0.974 ± 0.470
Roots not in contact with the sinus (*n* = 9)	19.602 ± 2.297	19.561 ± 2.396	−0.041 ± 0.253
